# A review of Dynamin 2 involvement in cancers highlights a promising therapeutic target

**DOI:** 10.1186/s13046-021-02045-y

**Published:** 2021-07-22

**Authors:** Delphine Trochet, Marc Bitoun

**Affiliations:** Centre de Recherche en Myologie, Sorbonne Université, Inserm, UMRS 974, Institut de Myologie, F-75013 Paris, France

**Keywords:** Dynamin 2, Cancer, Dynamin overexpression, Metastasis, Cell proliferation, Cell migration, Therapy

## Abstract

Dynamin 2 (DNM2) is an ubiquitously expressed large GTPase well known for its role in vesicle formation in endocytosis and intracellular membrane trafficking also acting as a regulator of cytoskeletons. During the last two decades, DNM2 involvement, through mutations or overexpression, emerged in an increasing number of cancers and often associated with poor prognosis. A wide panel of DNM2-dependent processes was described in cancer cells which explains DNM2 contribution to cancer pathomechanisms. First, DNM2 dysfunction may promote cell migration, invasion and metastasis. Second, DNM2 acts on intracellular signaling pathways fostering tumor cell proliferation and survival. Relative to these roles, DNM2 was demonstrated as a therapeutic target able to reduce cell proliferation, induce apoptosis, and reduce the invasive phenotype in a wide range of cancer cells in vitro. Moreover, proofs of concept of therapy by modulation of DNM2 expression was also achieved in vivo in several animal models. Consequently, DNM2 appears as a promising molecular target for the development of anti-invasive agents and the already provided proofs of concept in animal models represent an important step of preclinical development.

## Background

Dynamin 2 (DNM2) belongs to a superfamily of large GTPases including three classical dynamins and several dynamin-like proteins sharing propensity to self-assemble into polymeric structures [1]. DNM2 is ubiquitously expressed and is encoded by the *DNM2* gene producing four major splice isoforms using a combination of two alternative splice sites [2]. In addition, a muscle-specific isoform was identified [3]. DNM2 is a central actor of many membrane remodeling processes including clathrin-mediated endocytosis [4], clathrin-independent endocytosis [5, 6], coat-independent endocytosis [7, 8], intracellular vesicle trafficking [9–13], and exocytosis [14]. For its function of vesicle formation, DNM2 oligomerizes in a helical structure around the neck of nascent vesicles [4], and GTP hydrolysis leads to modifications in the helical structure associated with the release of the vesicles [15]. A function of regulator of actin [16–18] and microtubule [19, 20] cytoskeletons is now well recognized for DNM2 which sustains, in particular, its participation to mitosis and cell cycle progression [8, 21–23]. Heterozygous mutations in the *DNM2* gene are responsible for autosomal dominant forms of three human diseases, i.e. centronuclear myopathy [24], Charcot-Marie-Tooth disease [25], and hereditary spastic paraplegia [26] and one homozygous mutation was shown to cause a lethal congenital syndrome [27]. *DNM2* has also been described as a susceptibility gene for late-onset Alzheimer disease [28]. In addition, numerous studies are now available involving DNM2 in several cancers. The purpose here is to review involvement of DNM2 in pathophysiology of cancers and highlight the opened perspectives in medicine using DNM2 as a therapeutic target in these conditions.

## DNM2 deregulation in cancers

Acute lymphoblastic leukaemia (ALL) is the most common malignancy in B cells, immature T lymphocytes or lymphoid progenitors. Somatic heterozygous mutations in the *DNM2* gene were identified in children affected by early T-cell precursor acute lymphoblastic leukaemia (ETP-ALL), a form of ALL associated with a high risk of treatment failure [29]. In this study, *DNM2* mutations have been identified in 17 patients (13 out of 64 ETP patients and 4 out of 42 non ETP ALL patients) including 2 cases with biallelic mutations. The 19 mutations include 3 frameshifts, 3 splice site mutations, 6 missense mutations, 2 nonsense mutations, and 5 in frame deletions or insertions not found in centronuclear myopathy, Charcot-Marie-Tooth disease and hereditary spastic paraplegia. Thereafter, 4 other *DNM2* mutations (1 nonsense and 3 missense) have been described in adult T cell ALL patients extending the DNM2 involvement in ALL [30]. Clinical analysis of this small cohort of patients suggested that *DNM2* mutations are associated with a poor prognosis.

In addition to the *DNM2* mutations, an increased DNM2 expression was also identified in several cancer types (summarized in Table 1). DNM2 expression is increased in ALL, especially in B-ALL in which overexpression is associated with leukaemia cell proliferation and a poor prognosis [31]. DNM2 overexpression was also demonstrated in stem cells and progenitor cells of chronic myeloid leukemia (CML), i.e. a clonal myeloproliferative disorder originating from hematopoietic stem cells [32], in multiple ovarian cancer data sets [33], in bladder tumors in which the overexpression is correlated with the grade progression [34], in papillary thyroid cancer in which high DNM2 expression is associated with unfavourable prognosis (tumor recurrence, overall survival rate, distant metastases) [35], and in prostate cancer in which high DNM2 expression is associated with higher aggressiveness (Gleason score) and mortality [36, 37]. DNM2 overexpression was also reported in cervical cancer. In this cancer, the initial transformation of normal epithelium to preneoplastic cervical intraepithelial neoplasia (CIN) may be followed by a transformation to invasive cancer. DNM2 is not expressed in normal cervical epithelium but is overexpressed in preinvasive low grade lesions (CIN grade 1). With cancer progression, DNM2 expression decreases through an unidentified mechanism leading to low or no expression in high-grade lesions (CIN grades 2 and 3) especially, in case of deep tumor invasion and lymph node metastasis [38, 39]. These results establish DNM2 expression as a biomarker in grading of CIN, with a negative correlation between DNM2 expression and the severity of lesions, and suggest a crucial impact of DNM2 overexpression in the earliest steps of neoplasia of cervix. Both expression and localization of DNM2 were studied in tumor and adjacent normal tissue from 113 patients affected by several types of breast cancers [40]. All these breast tumor tissue samples exhibit a higher DNM2 expression compared to normal tissues. Among these cases, cytoplasmic overexpression was correlated with a specific cancer sub-type (invasive ductal carcinoma) whereas membranous DNM2 staining was associated with vascular invasion, an indicator of aggressiveness of breast cancer. Intriguingly, an increased expression of DNM2 was also noticed in the nucleus which is positively correlated with the histological grade and the tumor stage. This non-conventional nuclear localization also suggests a gain-of-function in the nucleus which needs to be deepen explored. In a particular sub-group of breast cancer, i.e. the triple negative breast cancers, defined by absence of estrogen receptor, progesterone receptor and without overexpression or amplification of EGF receptor 2, the level of DNM2 overexpression was negatively correlated with positive chemotherapy outcome [41]. The greater relapse rate in the patients exhibiting the higher DNM2 expression in tumor was attributed to the role of DNM2 in homology-directed repair of DNA, involving a DNM2 mediated trafficking of the RAD51 recombinase, which may mitigate over time the benefit of DNA-damaging chemotherapy in case of DNM2 overexpression. Finally, DNM2 expression was also shown to be markedly upregulated in tumors and metastases of patients affected by pancreatic ductal adenocarcinoma and this overexpression contributes to lamellipodia extension, cell migration and invasion [42]. In addition, patients with the highest levels of DNM2, associated with high level of α-actinin 4, had lower mean survival times compared to patients with lower expression [43]. Altogether, these data highlight the frequent up-regulation of DNM2 in cancers which can be used as a marker of poor prognosis.

**Table 1:** Summary of cancers with Dynamin 2 mutations or overexpression

**Type of cancer**	**DNM2 defect**	**Phenotypic association**	**References**
Acute Lymphoblastic leukemia (adult and children)	Somatic Mutations	• High risk of treatment failure • Poor prognosis (adult)	[29, 30]
Acute Lymphoblastic leukemia	Overexpression	• Cell proliferation • Poor prognosis	[31]
Chronic myeloid leukemia	Overexpression		[32]
Ovarian	Overexpression		[33]
Bladder	Overexpression	• Correlated with grade progression	[34]
Papillary Thyroid cancer	Overexpression	• Poor prognosis	[35]
Prostate	Overexpression	• Aggressiveness • Mortality	[36, 37]
Cervical	Overexpression	• Biomarker in grading neoplasia	[38, 39]
Breast	Overexpression	• Relapse to chemotherapy in triple negative breast cancer • Cytoplasmic DNM2 in invasive ductal carcinoma • Plasma membrane DNM2 ➔ aggressiveness • Nuclear DNM2 staining correlated with tumor stages	[40, 41]
Pancreas	Overexpression	• Cell migration and invasion • Lower man survival times	[42]

## Mechanisms responsible for DNM2 overexpression in cancers

**Fig. 1:**
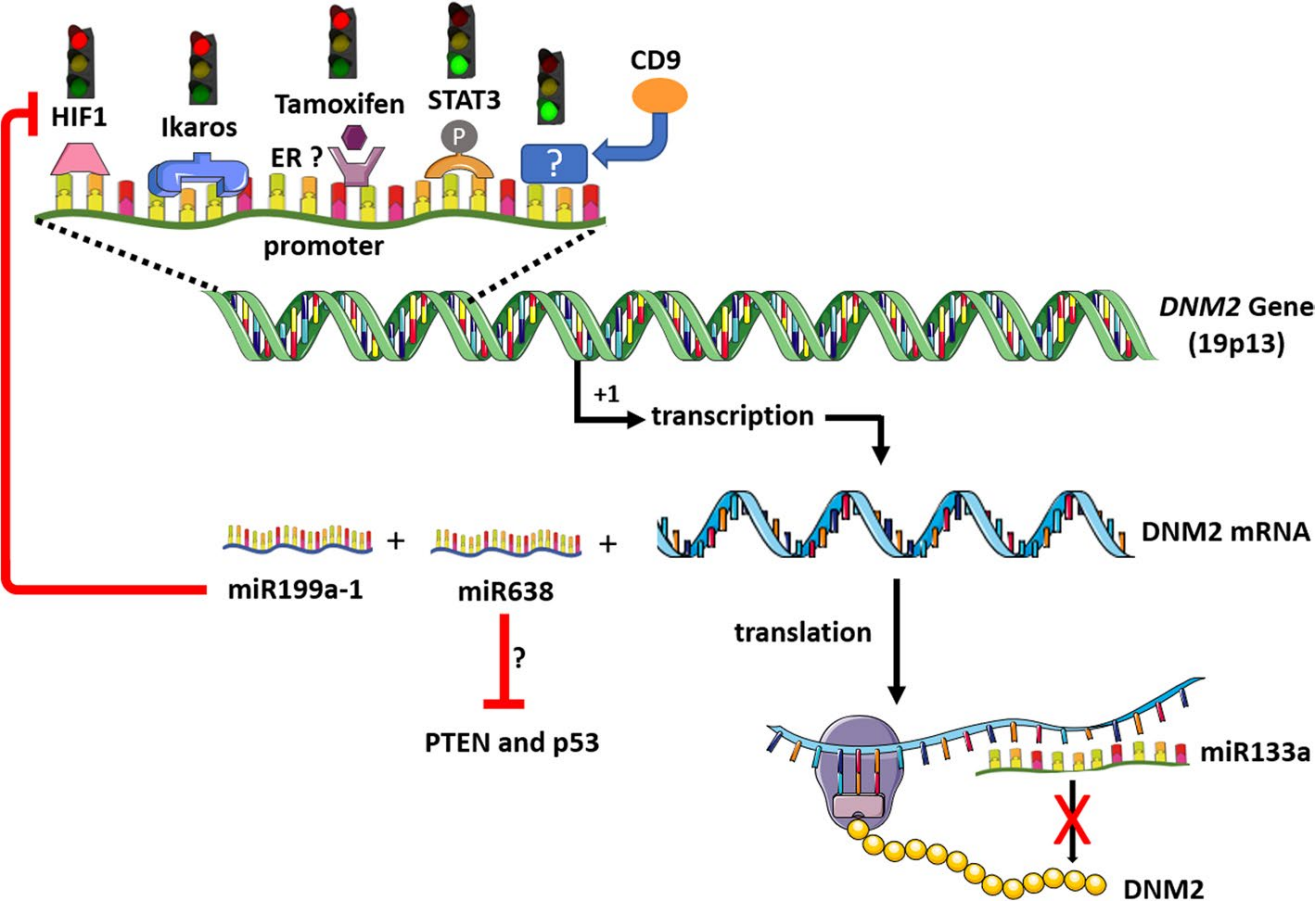
Regulation of DNM2 expression. CD9: CD9 tetraspanin. ER: estrogen receptors. HIF1: hypoxia-induced factor 1. P: phosphorylation of STAT3. PTEN: phosphatase and tensin homolog deleted on chromosome 10. STAT3: signal transducer and activator of transcription 3. Red light: inhibition of DNM2 transcription. Green light: Activation of DNM2 transcription. The figure was built using the Servier medical art database

The mechanisms underlying DNM2 overexpression in cancers have not been extensively studied and remains an important open question. Of note, Ikaros was shown to bind to the *DNM2* promoter in B cells [31] (Fig. 1). Ikaros is a DNA-binding zinc finger protein essential for normal haematopoiesis and immune development and acting as a tumor suppressor gene in acute B- and T-cell ALL. The correlation between low level of Ikaros mRNA and high level of DNM2 mRNA in ALL cells reinforces the hypothesis that Ikaros depletion may explain the high DNM2 expression in this pathological condition [31]. In addition, it was shown in ovarian cancer cell lines that DNM2 levels are transcriptionally down-regulated by the hypoxia-induced factor 1 (HIF-1) which directly binds to the *DNM2* promoter [44]. Consequently, HIF-1 inhibition may participate to DNM2 overexpression. The finding that DNM2 overexpression conversely reduced HIF-1α expression [44] may create a vicious circle maintaining a high level of DNM2 transcription. Finally, a regulation of DNM2 expression by CD9, a member of the tetraspanin family playing important functions in signal transductions from the plasma membrane, was demonstrated in pancreatic cancer cell lines [45]. In these cells, overexpression of CD9 led to the upregulation of the DNM2 expression by an unsolved molecular pathway.

Other potential regulators of *DNM2* transcription in cancers can be inferred from studies relative to another disease in which deleterious DNM2 overexpression occurs. The myotubular myopathy is the severe X-linked form of centronuclear myopathy due to mutations in the *MTM1* gene encoding Myotubularin 1. Patients and mouse model of myotubular myopathy exhibit an increased DNM2 expression and a forced DNM2 reduction restores phenotypes and lifespan in a mouse model of the disease [46, 47]. A recent study identified overexpression and activation by phosphorylation of the transcription factor STAT3 (signal transducer and activator of transcription 3) as responsible for the DNM2 upregulation in this pathological condition [48]. STAT3 is a key regulator of cell proliferation, survival and apoptosis and is constitutively activated in most human cancers including prostate, pancreas, breast cancers and leukaemia [49–51]. It is tempting to speculate that STAT3 activation is involved in DNM2 upregulation in cancers and that DNM2 overexpression may be an effector of features associated with abnormal STAT3 activation in cancers such as tumor growth and metastasis leading to poor prognosis [49–51]. In addition, the demonstration that tamoxifen, known to bind estrogen receptors, normalizes DNM2 expression and improves the phenotype of the *Mtm1*-deficient mouse model suggests that estrogen receptors may be involved in the regulation of the DNM2 expression [52].

Besides the above-mentioned transcriptional regulations of the *DNM2* gene, post-transcriptional mechanisms may be also involved in DNM2 upregulation in cancers. DNM2 is a target of miR-133a-1 and miR-133a-2 (Fig. 1) and silencing of miR-133a in mouse induces an increase in DNM2 in skeletal muscle and a centronuclear myopathy phenotype [53]. It is tempting to speculate that downregulation of miR-133a, as reported in prostate, pancreas and bladder cancers [54], may participate to the DNM2 deregulation in cancers.

## Involvement of DNM2 dysfunction in cancer pathophysiology

**Fig. 2:**
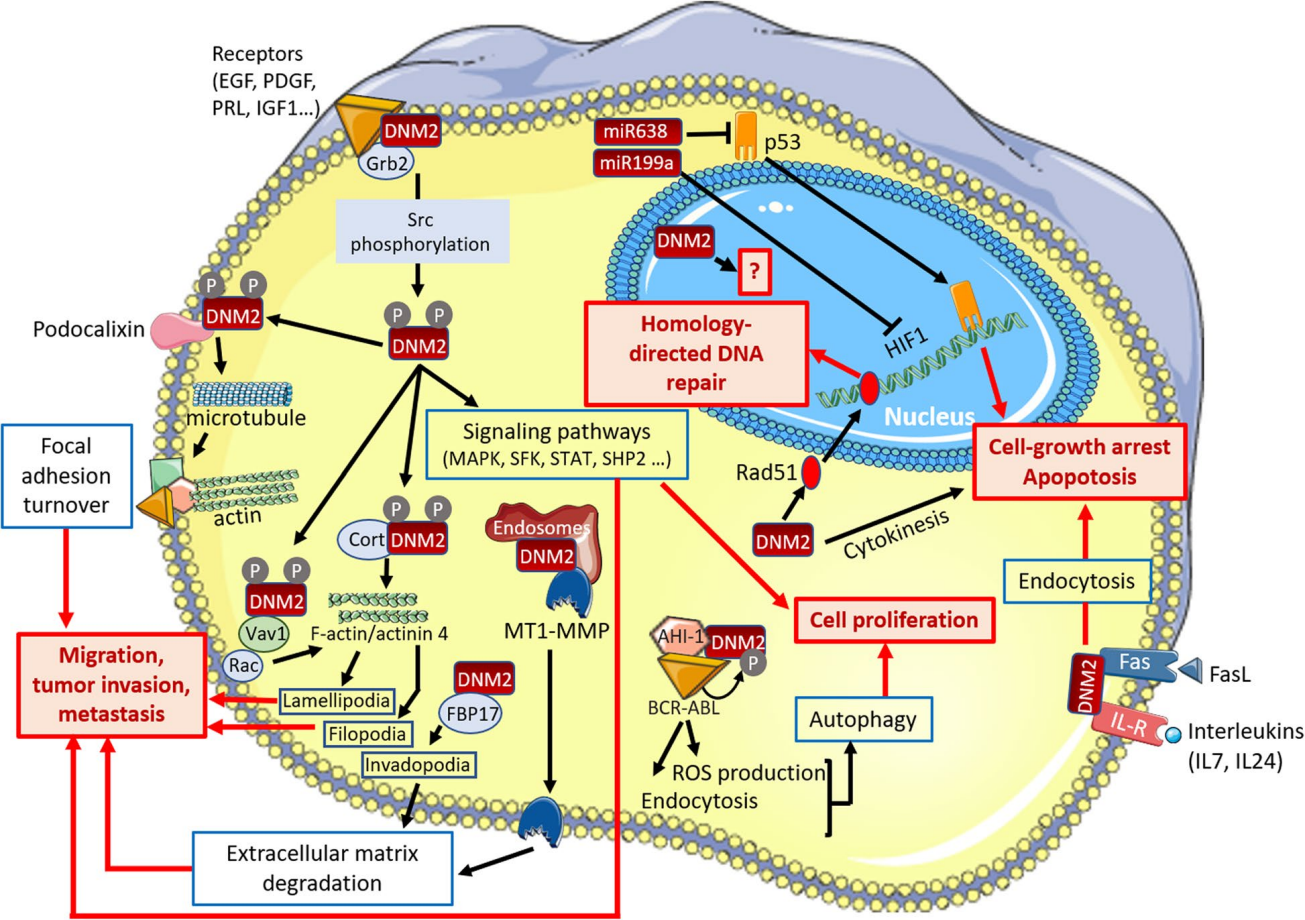
DNM2-dependent processes in tumor cells. AHI-1: Abelson helper integration site-1. BCR-ABL: product of the BCR-ABL fusion gene. Cort: Cortactin. DNM2: Dynamin 2. FasL: Fas Ligand. FBP17: Formin Binding Protein 17. HIF1: hypoxia-induced factor 1. IL-7: interleukin 7. IL-24: interleukin 24. MAPK: mitogen-activated protein kinase. MT1-MMP: membrane type 1 matrix metalloproteinase. P: phosphorylated residue in DNM2. PRL: prolactin. ROS: reactive oxygen species. SFK: Src-family kinase. SHP2: tyrosine-protein phosphatase non-receptor type 11. STAT3: signal transducer and activator of transcription 3. The miR199a and miR638 are both produced from the *DNM2* gene. The figure was built using the Servier medical art database

Several DNM2-dependent pathways have been identified in cancer cell lines and may be involved in cancer pathomechanisms. These mechanisms, summarized in Fig. 2, may be classified in 2 groups: mechanisms promoting cell migration and metastasis, and deregulation of intracellular signaling pathways linked to cell proliferation and survival.

### Migration, invasion and metastasis

Cancer cell migration requires formation of actin-based specialized structures such as lamellipodia and filopodia. By its role in the global organization of the actomyosin cytoskeleton through Rac- and cortactin-dependent processes [55–59], DNM2 appears as an key factor for formation and maintenance of these plasma membrane protrusions providing invasive behaviour of cancer cells. Several molecular pathways underlying the DNM2 involvement in cell migration and invasion have been identified in cancer cells. DNM2 mediates the Platelet Derived Growth Factor Receptor α (PDGFRα)-stimulated growth and invasion of glioblastoma cells through the formation of a Src-phosphorylated DNM2-PDGFRα-tyrosine-protein phosphatase non-receptor type 11 (SHP2) complex [60]. In pancreatic adenocarcinoma cell lines, the invasive phenotype was associated to the interaction between DNM2 and Vav1, a Rac1 guanine exchange factor abnormally expressed in pancreatic cancer [61]. In these cells, the physical DNM2-Vav1 interaction protect Vav1 from lysosomal degradation and then promotes activation of Rac1, a small GTPase known to regulate actin dynamics and branching, and the subsequent formation of lamellipodia and invasive cell migration [61]. Like for glioblastoma, importance of phosphorylation of DNM2, at known Src phosphorylation sites, was also demonstrated in migration of pancreatic cancer cell line as overexpression of a phospho-deficient DNM2 mutant is inefficient to promote lamellipodia formation and motile phenotype [42]. In addition to this DNM2 impact on actin cytoskeleton, its impact on microtubule cytoskeleton was demonstrated for migration of pancreatic cancer cells [62]. Indeed, through a direct interaction with Podocalyxin, a plasma membrane glycoprotein abnormally expressed in several cancers, DNM2 regulates the microtubule dynamics leading to reduction of focal adhesions promoting cell migration [62].

On the other hand, plasticity of cell environment, especially through modification of the extracellular matrix, plays an important role in tumor cell invasion and DNM2 was also implicated in this process. DNM2 activity is required for extracellular matrix degradation by invasive tumor cells at invadopodia, another specialized actin-based plasma membrane protrusion [63]. Formation of invadopodia and invasive phenotype of bladder tumor cells involve Formin Binding Protein 17 (FBP17) belonging to the family of formin-binding proteins which regulate the formin-dependent actin assembly [64]. FBP17 interacts with DNM2 in these cells and may then recruit DNM2 to the invadopodia. Extracellular matrix remodelling mediated by invadopodia depends on matrix-degrading proteases such as the secreted and membrane-anchored matrix metalloproteinases including the membrane type 1 matrix metalloproteinase (MT1-MMP). DNM2 provides the proper delivery from late recycling endosomes of MT1-MMP to the invadopodia of breast cancer cells and is required for their ability to degrade matrix [65]. Of note, MT1-MMP is overexpressed in 25% of triple-negative breast cancers [65], the type of aggressive and highly proliferative breast tumors in which DNM2 is also overexpressed [41]. Concomitant overexpression of both DNM2 and MT1-MMP may synergize to increase aggressiveness of breast tumors.

In summary, DNM2 directly impacts tumor cells invasiveness and consequent metastasis by stabilizing distinct actin-based structures involved in cell migration, by remodelling extracellular matrix through metalloproteinase delivery and by promoting focal adhesions disassembly. The regulation of the actin dynamics as the main DNM2 function involved in metastasis was confirmed by the role highlighted for the DNM2-α-actinin 4 complex in the lamellipodia-mediated migration and invadopodia-mediated matrix degradation in pancreatic ductal adenocarcinoma, one of the most aggressive cancer associated with high rates of metastasis [43].

### Cell proliferation and survival

DNM2 is the main interactor of the ubiquitously expressed adapter Growth factor receptor-bound protein 2 (Grb2) in hepatocarcinoma cells, suggesting that various signal transduction pathways involving Grb2 may be impaired in case of DNM2 deregulation in cancer cells [66]. Over-activation of such signal transduction pathways, requiring a DNM2-dependent receptor internalization for their downstream signaling, may be deleterious by increasing tumor cell proliferation and survival as suggested in breast cancer and leukemia cells. In breast cancer cells, the association between prolactin exposure and development of invasive breast cancer is widely accepted. Interestingly, the ligand-induced prolactin receptor endocytosis, required for the activation of the downstream Src family kinase-mediated signaling cascade linked to cell proliferation, was shown to be DNM2-dependent [67]. In the same line, prolactin also enhances Insulin growth factor 1 (IGF1)-induced phosphorylation of IGF1-receptor increasing its AKT and ERK1/2 downstream signaling associated with proliferation, survival and invasion of breast cancer cells. It was demonstrated in breast cancer cells that DNM2-dependent endocytosis of the IGF1-receptor is required for the prolactin-induced increase of IGF1-receptor phosphorylation inducing IGF1-receptor signaling [68]. In chronic myeloid leukemia cells, a complex formed by DNM2, Abelson helper integration site-1 (AHI-1) and the fusion oncogene BCR-ABL leads to activation of DNM2 through its phosphorylation by the BCR-ABL tyrosine kinase [32]. This aberrant complex was involved in the increase in clathrin-mediated endocytosis, reactive oxygen species (ROS) production and autophagy in the chronic myeloid leukemia stem and progenitor cells [32] possibly at the origin of the cell survival, genomic instability and resistance to treatment. An increase in endocytosis following DNM2 overexpression may also help cancer cells to protect themselves to complement-dependent necrotic cell death as suggested by the DNM2-dependent internalization of complement complexes from the plasma membrane in leukemia cells [69].

In some other conditions, it is more difficult to anticipate the potential functional consequences of DNM2 overexpression. For example in melanoma cells, DNM2 regulates plasma membrane content of the Fas receptor which plays an important role in programmed cell death when bound to Fas ligand [70, 71]. Similarly, the DNM2-dependent endocytosis of the Interleukin 24 (IL-24) with its receptor is required in prostate cancer cells for the tumor suppressor action of the IL-24 (also called MDA-7) [72]. In these two examples, overexpression of DNM2 may reinforce Fas ligand-induced apoptosis or the cancer-specific cell killing activity of IL-24. alternatively, the DNM2 overexpression in these cells may reduce by endocytosis the basal content of Fas and IL-24 receptor in absence of ligands and consequently reduce the beneficial signaling pathways when Fas and IL-24 arrived.

DNM2 overexpression may have also indirect deleterious impacts through concomitant upregulation of miRNA located at the *DNM2* locus as already demonstrated for the miR199a-1 in the pathophysiology of the myotubular myopathy [48]. This hypothesis may be of particular interest for the miR-638 located in the intron 1 of *DNM2* and known to target two of the main tumor suppressor genes, i.e. PTEN (phosphatase and tensin homolog deleted on chromosome 10) and p53 considered as the ‘‘guardian of the genome’’ [33]. A miR-638 upregulation in DNM2 overexpressing cells may reduce expression of PTEN and p53 (Fig. 1) and then deregulate their specific signaling pathways controlling cell growth and survival, DNA repair, cell-cycle arrest, and apoptosis.

Finally, only one study addressed the functional effects of the *DNM2* mutations and their contribution to the T-ALL pathogenesis [73]. This study suggested that *DNM2* mutations affect the clathrin-mediated endocytosis through a dominant-negative effect. By this pathomechanism, *DNM2* mutations increase the plasma membrane content of Interleukin 7 (IL-7) receptor in pre-leukemic thymocytes leading to enhancing IL-7 signaling and development of more immature T-ALL.

## Therapeutic developments

Benefit of DNM2 inhibition was largely documented for almost all the DNM2-related phenotypes in tumor cells in vitro. The function of the DNM2 in cytokinesis was targeted in order to influence the cell cycle progression and reduce proliferation of tumor cells. With this objective, pharmacological inhibitors of DNM2 [74, 75] were used to induce cytokinesis failure at the membrane abscission step leading to growth arrest and death of cervical, lung, and leukaemia cancer cell lines [76–78]. This induced cancer cell death following cytokinesis failure results from the intrinsic caspases 3 and 9-mediated apoptotic pathway and is more efficient in cancer cell lines harbouring low level of the anti-apoptotic Bcl-2 and Mcl-1 proteins [79]. Interestingly, nontumorigenic fibroblasts treated with DNM2 inhibitors appear less sensitive to cell death than cancer cells [76, 77]. A benefit a DNM2 inhibition to reduce cell proliferation and/or induce apoptosis was confirmed in a wide range of cancer cells including cervical epithelial cancer cells [80], prostate cancer cells [36, 42], non-small-cell lung cancer cells [81], glioblastoma cells [82], chronic myeloid leukemia cells [32], B- and T-ALL cells [31] and hepatocellular carcinoma cells [83]. The similar benefit using pharmacological DNM2 inhibitors [74, 75, 82, 84–88] or DNM2 gene silencing demonstrates the requirement of the DNM2 GTPase activity in the phenotypes occurring in these cancer cells. In hepatocellular carcinoma cells, dynamin inhibition accelerates degradation of c-Met, a tyrosine kinase receptor involved in hepatocellular carcinoma development and progression, and decreases the c-Met downstream signaling [83]. DNM2 inhibition can also reduce the invasive phenotype of tumor cells in vitro. The benefit of pharmacological inhibitors [75, 84, 85, 89] or DNM2 mRNA silencing on cell invasion in prostate, lung, bladder, and pancreatic cancer cells is provided through an impact on actin dynamics and formation of the actin-based protrusions (lamellipodia, filopodia, and invadopodia) and/or stabilizing focal adhesion which impede invasive behaviour [36, 42, 43, 57, 58, 61, 62, 89]. In melanoma and breast cancer cell lines, DNM2 inhibition impacts cell invasion by reducing extracellular matrix remodelling and the number of degrading cells [63, 65]. DNM2 inhibition was also achieved by targeting the *DNM2* gene transcription. Indeed, due to the role of Ikaros in DNM2 expression in ALL cells, effect of 4,5,6,7-Tetrabromobenzotriazole (TBB), an enhancer of Ikaros tumor suppressor activity, was investigated. Interestingly, TBB increased Ikaros binding to the DNM2 promoter and inhibited expression of DNM2 mRNA in ALL cells [31]. A better understanding of the mechanisms underlying the DNM2 overexpression in the different types of cancers will be helpful for developing similar therapeutic approaches.

Importantly, the proof of concept of therapy by modulating DNM2 expression was also achieved in vivo in several animal models (Table 2). In order to assess whether lowering DNM2 levels would increase sensitivity to chemotherapy, breast cancer cells expressing an inducible shRNA to silence DNM2 by RNA interference were implanted into mammary fat pads of mice. Reduced expression of DNM2 significantly improved tumor response to cyclophosphamide, a drug widely used in chemotherapy of breast cancers, leading to reduced tumor volume [41]. A proof of concept was also done using pancreatic cancer cells in vivo [42]. Orthotopic injection of cells overexpressing DNM2 promotes tumor cells dissemination distal from the injection area compared to mice injected with pancreatic cancer cells with basal DNM2 expression. Inhibition of DNM2 by overexpressing phospho-deficient mutant in the injected pancreatic cancer cells drastically reduces the number of large tumors outside the injection site. A third study in which prostate cancer cells were injected in prostate of mice showed that tumor weight and lymph node metastases are reduced when DNM2 is inhibited by RNA interference in the injected cells [36]. A similar result was reported for the PDGFRα-stimulated glioma cell growth and invasion in brain of mice [60]. In this case, injection of DNM2-depleted cells is associated with decrease in tumor cell proliferation and increase in apoptosis. Proofs of concept were also achieved using pharmacological compounds. In a prostate cancer mouse model in which formation of subcutaneous tumors are induced after injection of human prostatic adenocarcinoma cells, intratumoral injection of a DNM2 inhibitor (N’-[4-(dipropylamino)benzylidene]-2-hydroxybenzohydrazide, DBHA) rapidly reduces tumor size without apparent adverse effect [89]. In the same line, continuous delivery of the CyDyn4-36 DNM2 inhibitor by subcutaneous osmotic pumps reduces the size of established tumors formed by prior intracranial injection of glioma stem cells [82] and intraperitoneal injection of the Dynole 34.2 DNM2 inhibitor leads to progressive exhaustion of pre-leukemia stem cells in a mouse model of acute leukemia [90]. Dynole 34.2 also reduces the number of leukemic cells in the bone marrow and spleen of mice after T-ALL and acute myeloid leukemia cells xenografts [90]. DBHA, Dynole 34.2 and CyDyn4-36 represent a new generation of DNM2 inhibitors opening the way for future clinical use. DBHA, is an analogue of the non-competitive inhibitor dynasore, identified through a screening for inhibitors of dynamin GTPase activity, which suppress actin dynamics and cancer cells migration more efficiently with less cytotoxicity compared to dynasore [85, 89]. Dynoles are also non-competitive inhibitors of dynamin GTPase activity [75] and the Dynole 34–2 was shown as the most effective at causing cancer cell death [77]. CyDyn4-36 is a next-generation Dynole designed to be more brain penetrant through a reduction in polar surface area and the number of hydrogen bond donors and acceptors [82]. Altogether, these data highlight Dynamin 2 as a promising molecular target for the development of anti-invasive agents and the proof of concept of therapy already achieved by reducing DNM2 expression in animal models is an important step of preclinical development.

**Table 2:** Proof of concept in animal models for therapeutic benefit of reduction of DNM2

Type of cancer	Animal model	Approach for DNM2 reduction	Read out	Reference
Prostate	Implantation of tumor cells (PC3, LNCaP, and C4-2) in prostate of male SCID mice	Stable expression of DNM2-siRNA or scrambled-siRNA in injected cells	9 weeks after cell injection: • Decrease in tumor weight • Reduction of number of lymph node metastases (for the PC3 cells able to induce metastases)	[36]
Prostate	Subcutaneous injection of PC3 cells in athymic mice	Pharmacological inhibitor (DBHA). Intratumoral injection in tumors of 7–13 mm	• Reduction of the tumor volume at day 4 and day 8 after injection (vs vehicle injected tumors) • No apparent toxic effect at the necropsy (day 8)	[89]
Pancreas	Implantation of tumor cells overexpressing DNM2 or phospho-deficient DNM2 (PxPC-3) in pancreas of nude mice	Stable expression of WT DNM2-GFP or phospho-deficient DNM2-GFP in injected cells	2 weeks after cell injection: • Comparable size of primary tumor • Expression of the phospho-deficient mutant limits the distal dissemination of tumor cells from the injection area (vs WT DNM2-expressing cells) 8 weeks after cell injection: • Similar volume of the primary pancreas tumors • Large tumors in the body cavity • Expression of the mutant DNM2 decrease the number of large intestinal tumors vs WT DNM2-expressing cells • No liver tumors after injection of cells expressing the mutant (which occurs in 3 of 18 mice injected with cells overexpressing WT DNM2)	[42]
Breast	Injection of tumor cells expressing inducible DNM2 shRNA (MDA-MB-231-BR3) into mammary fat pads of nude mice	Doxycycline-inducible shRNA against DNM2 and control shRNA in injected cells	• No decrease in tumor volume alone • Improvement of the tumor volume reduction induced by chemotherapy by cyclophosphamide	[41]
Glioblastoma	Injection of tumor cells (LN444/PDGF-A) into the brain of mice	DNM2-siRNA or control-siRNA in injected cells	8 weeks after cell injection: • Suppression of the PDGFRα–stimulated glioma growth (tumor volume) and invasion (number of prodruded fingers from tumors) • Decrease in tumor cell proliferation • Increase in cell apoptosis	[60]
Glioblastoma	Injection of tumor cells (GSC#035 with stable expression of luciferase) into the brain of nude mice	Continuous release of a DNM2 inhibitor (CyDyn 4–36) for 14 days by subcutaneous osmotic minipumps once tumors were established	Luciferase in vivo imaging after 1, 4, 8, 11 and 14 days of treatment: • Reduction of tumor volume statistically significant from 11 days of treatment (vs vehicle treated mice)	[82]
Leukemia	6-week-old Lmo2^Tg^ mice	IP injection twice daily for 5 days on 2 consecutive weeks of a DNM2 inhibitor (Dynole 34–2)	After 2 weeks of treatment: • Reduction in the number of DN3a thymocytes • Decrease in pre-LSC frequency • Progressive exhaustion of pre-LSCs In non-tumour-bearing control mice: no detrimental effect of treatment on differentiated cells in the thymus and the bone marrow or the number of phenotypic bone marrow stem and progenitor cells	[90]
Leukemia	Injection of immature (ETP12) and mature (ALL8) T-ALL cell lines in mice	IP injection twice daily for 5 days on 2 consecutive weeks of a DNM2 inhibitor (Dynole 34–2). Treatment started when the average proportion of leukemic cells in the peripheral blood reached 1%	• Increased survival of treated mice 24 h after the last administration: • Reduction in leukemic cells in the peripheral blood, bone marrow and spleen • Inhibition of the abnormally activated IL-7 and NOTCH1 signaling pathways in leukemic cells	[90]
Leukemia	Injection of AML cell lines AML01-307 and AML18) in immunodeficient mice	IP injection twice daily for 5 days on 2 consecutive weeks of a DNM2 inhibitor (Dynole 34–2)	• Delayed onset of the disease • Increased survival of treated mice 24 h after the last administration: • Inhibition of IL-3, GM-CSF and SCF signaling pathways in leukemic cells • Less patient-derived AML cells in bone marrow and spleen of treated mice	[90]

*AML* Acute myeloid leukemia, *C4-2* Androgen-resistant variant of the LNCaP cells, *DBHA* N-[4-(dipropylamino)benzylidene]-2-hydroxybenzohydrazide, *DN3a thymocytes* Population of T-cell progenitors (CD4^−^ CD8^−^ CD44^−^ CD25^+^ CD28^low^) responsible for the preleukemic stem cells activity in the Lmo2^Tg^ mouse model of T-ALL, *GSC#035* Glioma stem cell line, *IP* Intraperitoneal, *Lmo2*^*Tg*^ Cd2-Lmo2-transgenic mouse model of T-cell acute lymphoblastic leukemia (T-ALL), *LN444/PDGF-A* Glioblastoma cell line expressing PDGF-A, *LNCaP* Androgen-responsive prostate cancer cell line, *MDA-MB-231-BR3* Triple-negative breast cancer cell line, *PC3* Invasive and androgen receptor negative prostate cancer cell line, *Phospho-deficient DNM2* Double mutant Tyrosine (231/597) Phenylalanine, *Pre-LSC* Preleukemic stem cells, *PxPC-3* Pancreatic epithelial tumor cell line, *SCID mice* Severe combined immunodeficiency mice, *siRNA* Short interfering RNA inducing DNM2 reduction through RNA interference, *WT* Wild-type

## Conclusions

Numerous studies are now available demonstrating the interest of DNM2 as a biomarker for prognosis of tumor invasion and metastasis, as exemplified for cervical cancers, or to predict therapy efficacy as shown in a sub-group of breast cancer. The available data strongly suggest that DNM2 participates to the maintenance of the necessarily favourable environment for tumor to grow, invade and migrate. Interestingly, modulation of DNM2 expression may prove a valuable therapeutic target with proof of concept achieved in vitro and in vivo. Molecules targeting DNM2 expression and/or activity may expand the panoply of anti-mitotic and anti-metastatic agents in cancer treatments. Therapeutic potential of DNM2-based approaches may be of particular interest to counteract metastasis which mark the transition from a benign tumor to a lethal, malignant cancer with dissemination of tumor cells. However, future therapeutic intervention should avoid to excessively reduce DNM2 expression for maintaining the beneficial aspects of DNM2 function in cancer cells as DNA repair already mentioned [41] or the metastasis suppressor function of NME proteins [91]. Indeed, when re-expressed or overexpressed in metastatic tumor cells, NME proteins suppress cell motility and migration in vitro and metastatic colonization in vivo through a mechanism requiring DNM2-dependent endocytosis [91]. The importance to preserve DNM2 expression is also highlighted by the reported analysis of 131 hepatocellular carcinoma showing that patients with low DNM2 expression displayed a significantly worse overall survival [92]. This study concludes that drastic DNM2 downregulation by siRNA or drug in hepatocellular carcinoma cell lines provides colony formation, migration and invasion of tumor cells by reducing the role of DNM2 as a negative regulator of epidermal growth factor (EGF) signaling through the endocytosis of the EGF receptor [92]. Overall, the challenge of future DNM2-based therapies will be to simultaneously maintain DNM2 positive roles and counteract its deleterious functions in tumor cells.

## Data Availability

Not applicable.

## Abbreviations

AHI-1: Abelson helper integration site-1
ALL: Acute lymphoblastic leukaemia
CML: Chronic myeloid leukemia
DNM2: Dynamin 2
ETP-ALL: Early T-cell precursor acute lymphoblastic leukaemia
EGF: Epidermal growth factor
FBP17: Formin Binding Protein 17
Grb2: Growth factor receptor-bound protein 2
HIF-1: Hypoxia-induced factor 1
IGF1: Insulin growth factor 1
IL-7: Interleukin 7
IL-24: Interleukin 24
MT1-MMP: Membrane type 1 matrix metalloproteinase
PDGFRα: Platelet Derived Growth Factor Receptor α
PTEN: Phosphatase and tensin homolog deleted on chromosome 10
ROS: Reactive oxygen species
SHP2: Tyrosine-protein phosphatase non-receptor type 11
STAT3: Signal transducer and activator of transcription 3
TBB: 4,5,6,7-Tetrabromobenzotriazole
